# Cardiopulmonary toxicity of peat wildfire particulate matter and the predictive utility of precision cut lung slices

**DOI:** 10.1186/1743-8977-11-29

**Published:** 2014-06-16

**Authors:** Yong Ho Kim, Haiyan Tong, Mary Daniels, Elizabeth Boykin, Q Todd Krantz, John McGee, Michael Hays, Kasey Kovalcik, Janice A Dye, M Ian Gilmour

**Affiliations:** 1 Curriculum in Toxicology, University of North Carolina at Chapel Hill, Chapel Hill, NC, USA; 2 Environmental Public Health Division, National Health and Environmental Effects Research Laboratory, U.S. Environmental Protection Agency, Research Triangle Park, NC, USA; 3 Air Pollution Prevention and Control Division, National Risk Management Research Laboratory, U.S. Environmental Protection Agency, Research Triangle Park, NC, USA; 4 Human Exposure Assessment Division, National Exposure Research Laboratory, U.S. Environmental Protection Agency, Research Triangle Park, NC, USA

**Keywords:** Wildfire, Peat fire, Particulate matter, Lung inflammation, Cardiac perfusion, Lung tissue slices

## Abstract

**Background:**

Emissions from a large peat fire in North Carolina in 2008 were associated with increased hospital admissions for asthma and the rate of heart failure in the exposed population. Peat fires often produce larger amounts of smoke and last longer than forest fires, however few studies have reported on their toxicity. Moreover, reliable alternatives to traditional animal toxicity testing are needed to reduce the number of animals required for hazard identification and risk assessments.

**Methods:**

Size-fractionated particulate matter (PM; ultrafine, fine, and coarse) were obtained from the peat fire while smoldering (ENCF-1) or when nearly extinguished (ENCF-4). Extracted samples were analyzed for chemical constituents and endotoxin content. Female CD-1 mice were exposed via oropharyngeal aspiration to 100 μg/mouse, and assessed for relative changes in lung and systemic markers of injury and inflammation. At 24 h post-exposure, hearts were removed for *ex vivo* functional assessments and ischemic challenge. Lastly, 8 mm diameter lung slices from CD-1 mice were exposed (11 μg) ± co-treatment of PM with polymyxin B (PMB), an endotoxin-binding compound.

**Results:**

On an equi-mass basis, coarse ENCF-1 PM had the highest endotoxin content and elicited the greatest pro-inflammatory responses in the mice including: increases in bronchoalveolar lavage fluid protein, cytokines (IL-6, TNF-α, and MIP-2), neutrophils and intracellular reactive oxygen species (ROS) production. Exposure to fine or ultrafine particles from either period failed to elicit significant lung or systemic effects. In contrast, mice exposed to ENCF-1 ultrafine PM developed significantly decreased cardiac function and greater post-ischemia-associated myocardial infarction. Finally, similar exposures to mouse lung slices induced comparable patterns of cytokine production; and these responses were significantly attenuated by PMB.

**Conclusions:**

The findings suggest that exposure to coarse PM collected during a peat fire causes greater lung inflammation in association with endotoxin and ROS, whereas the ultrafine PM preferentially affected cardiac responses. In addition, lung tissue slices were shown to be a predictive, alternative assay to assess pro-inflammatory effects of PM of differing size and composition. Importantly, these toxicological findings were consistent with the cardiopulmonary health effects noted in epidemiologic reports from exposed populations.

## Background

It is well recognized that short- and long-term exposures to airborne pollutants are associated with increased morbidity and mortality in the exposed population [1,2]. Furthermore, these same patterns have been noted during acute wildland fire episodes, and it is expected that as the scale and frequency of wildfires increase because of climate change, hospital admissions for respiratory infections, asthma, cardiovascular diseases, and heart failure will also rise in urban areas impacted by such events [3-5]. Most studies of the health effects of wildfire exposure are based on occupational and epidemiological observations, and there are only a few reports describing the toxicity of wildfire particulate matter (PM) under controlled laboratory conditions [3,6]. In addition, much of the work has focused on forest fires or domestic woodburning, while peat fires that occur in wetlands consisting of biomass and organic soils, have not been widely studied [7]. In general, compared to forest fires, which spread quickly with extremely intense flame and high temperature, peat fires propagate slowly without flame but emit huge amounts of smoke, and can last for months or even years [8-10]. Despite the potential public health threat from increased wildland fire emissions [9-12], it is not known if there are common bioactive components that are more harmful than constituents from regional and point source pollutants, and only a limited number of studies have directly evaluated the toxicity of wildfire smoke which varies according to fuel type, combustion conditions, and meteorology [13-15]. Interestingly, lipopolysaccharide (LPS, endotoxin) which exacerbates both respiratory and cardiovascular disease, is present in biomass emissions from sources including domestic woodsmoke, agricultural burning, and environmental cigarette smoke [16,17].

While there is a clear need for more toxicological information on particulate matter from smoke emissions, there is also concern that such testing could vastly increase the number of animals required. Since Russell and Burch [18] first proposed the concept of the 3Rs (reduction, refinement, and replacement), there have been increasing efforts to limit the number of animals used in toxicology research, and to develop *ex vivo*/*in vitro* alternatives. Organ tissue slices provide the most *in vivo*-like environment as they preserve almost all cell types and their potential interactions with the natural matrix [19-22]. In this context, lung tissue slices have been widely used to study metabolism, toxicology, pharmacology, and cellular signaling mechanisms for the past 25 years, and can be prepared from a variety of species including mice, rats, hamsters, guinea pigs, rabbits, horses, and humans [23-28].

Observational studies from the Eastern North Carolina peat fires in the summer of 2008 found that exposure to peak concentrations of the smoke (as estimated by aerosol optical density obtained from satellite imaging), was associated with increased emergency department (ED) visits for asthma-related respiratory complications and the incidence of heart failure [29]. It remains unclear however whether differences in the size fraction(s) of the smoke and/or chemical composition(s), may have influenced these specific health impacts. We have previously reported that coarse (2.5-10 μm) PM from near roadways or other urban areas evoke pulmonary inflammatory responses in mice, while fine (0.1-2.5 μm) and ultrafine (<0.1 μm) PM preferentially affect the cardiovascular system [30-32]. In this current study, we collected size-fractionated PM over two periods of the Eastern North Carolina peat fires in 2008 (smoldering and glowing stages), and performed chemical analyses and acute pulmonary and cardiac toxicity studies in mice. Additionally, tests were conducted in mouse lung tissue slices to determine if this system could predict the *in vivo* pulmonary response profile. To our knowledge, this is the first study to evaluate the cardiopulmonary toxicity of size-fractionated wildfire PM from different periods in both *in vivo* and *ex vivo* bioassay systems.

## Results

### Eastern North Carolina wildfire (ENCF) PM sampling conditions and state monitoring data

**Figure 1:**
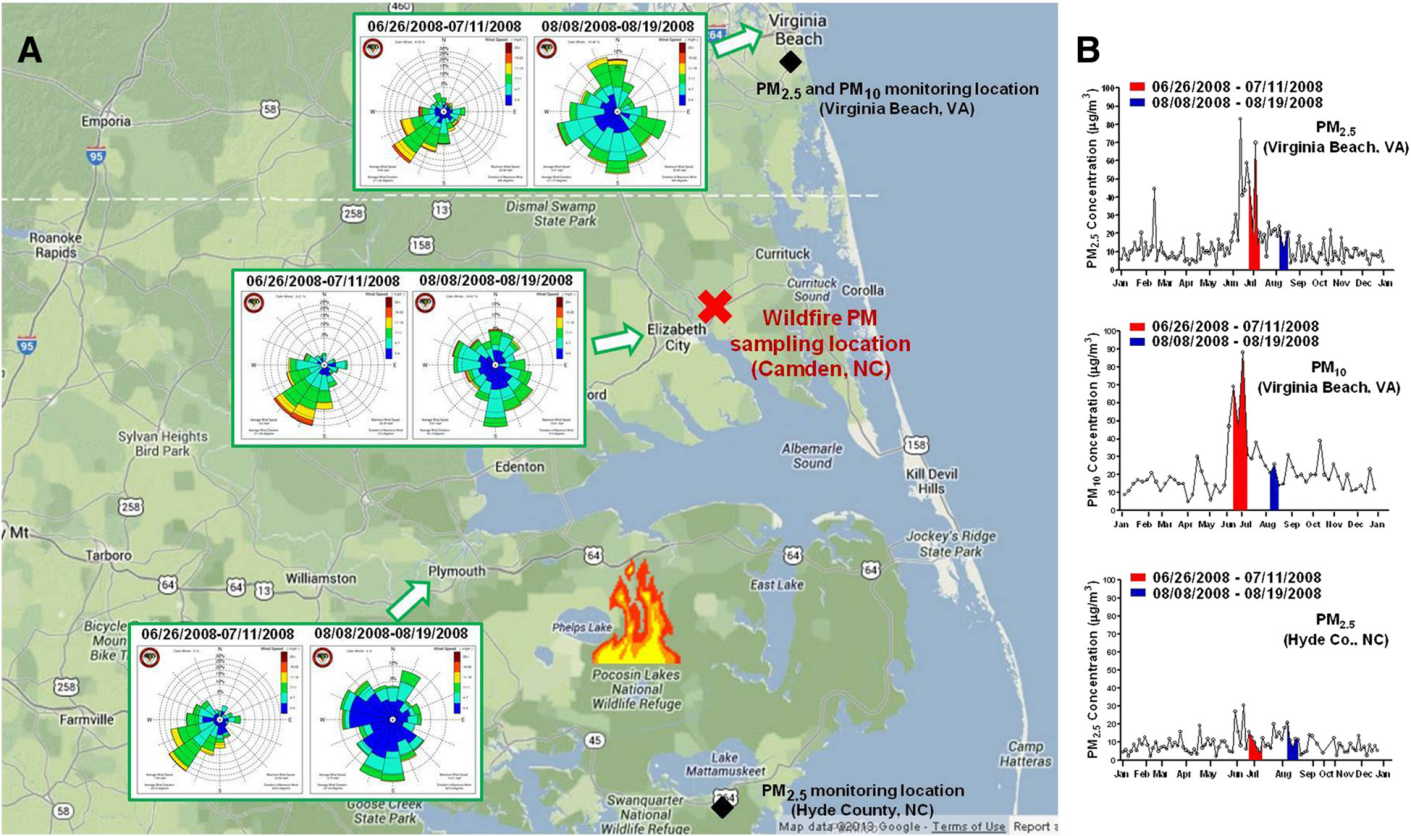
**Locations of peat fires and PM sampling site in Eastern North Carolina (A) and daily mean concentrations of PM**_**2.5 **_**and PM**_**10 **_**collected at Virginia Beach, VA and Hyde County, NC (B).** Peat-bog fires at the Pocosin Lakes National Wildlife Refuge in Eastern North Carolina started on 06/01/2008 and was 100% contained on 09/24/2008. Insert: wind rose diagram for the periods of ENCF-1 PM sampling (06/26/2008-07/11/2008) and ENCF-4 PM sampling (08/08/2008-08/19/2008) recorded at Plymouth, Elizabeth City and Virginia Beach, respectively. The wind rose diagram and PM monitoring data were obtained from the State Climate Office of North Carolina (http://www.nc-climate.ncsu.edu) and the U.S. EPA Air Data (http://www.epa.gov/airdata), respectively.

The peat-bog wildfire at the Pocosin Lakes National Wildlife Refuge started on 06/01/2008 as a result of a lightning strike. ENCF-1 PM samples were collected between June 16^th^ and July 11^th^ when the fire was actively smoldering, while ENCF-4 PM samples were collected between August 9^th^ and 18^th^ when the fire was glowing and nearly extinguished. Figure 1A illustrates the site of the wildfire, the “Chem-vol” sampling location, upwind (Hyde County) and downwind (Virginia Beach) PM monitoring stations as well as wind-rose diagrams recorded at Plymouth and Elizabeth City, NC and Virginia Beach, VA before, during, and after the sampling campaign. As can be seen, the wind blew predominantly in the direction of South-West during ENCF-1 PM sampling, while the wind direction diminished and was more southerly during ENCF-4 PM sampling. Figure 1B shows that concentrations of PM_2.5_ and PM_10_ downwind from the wildfire at Virginia Beach were approximately three times higher during ENCF-1 than ENCF-4, while concentrations of PM_2.5_ at the upwind Hyde County site were within the normal range for both periods.

### PM characteristics

**Figure 2:**
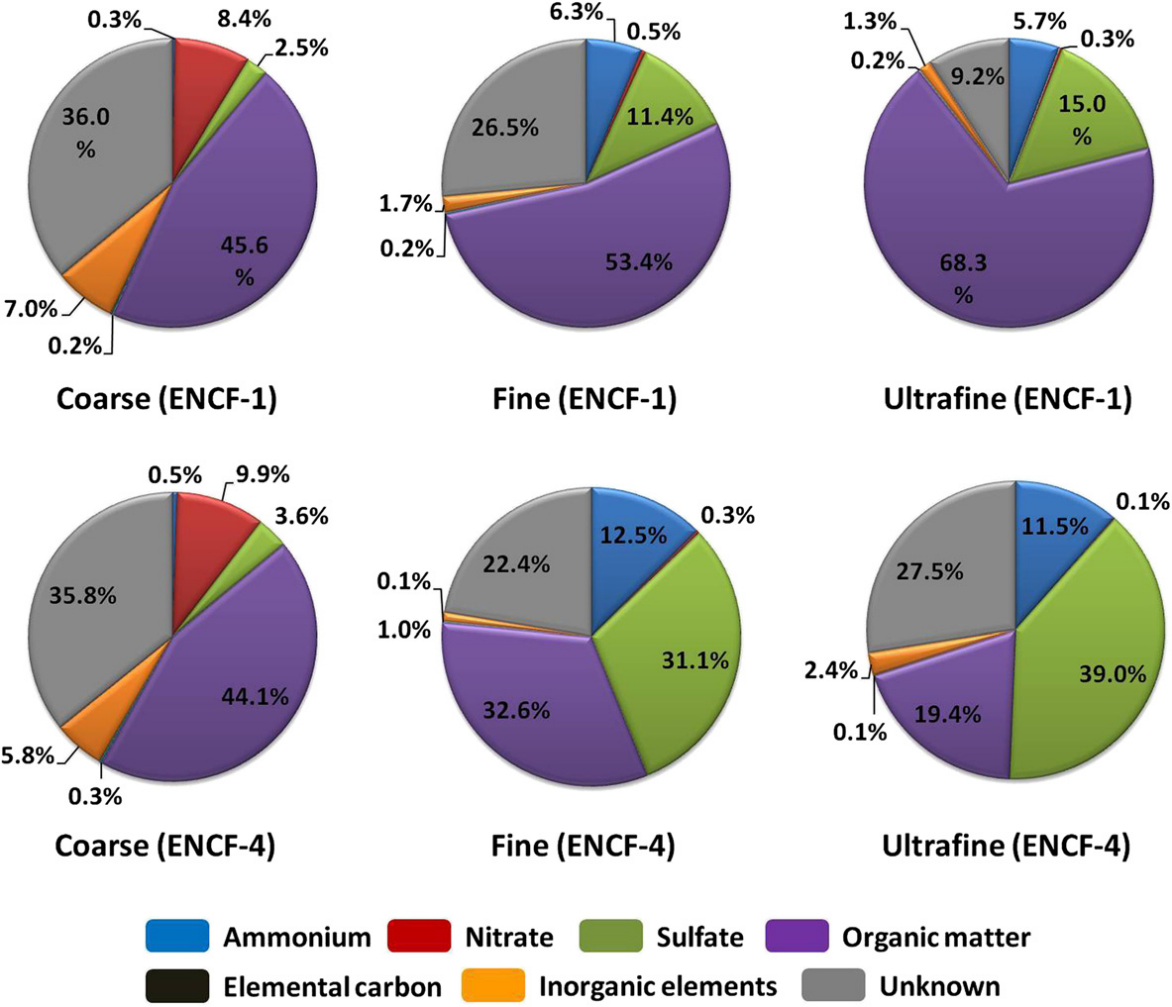
Mass balance of major classes of chemical components in each fraction of ENCF-1 and ENCF-4 PM.

Table 1 summarizes collected mass (daily average), chemical and endotoxin compositions in size-fractionated PM from the two sampling periods. Consistent with the higher PM concentrations from the state monitoring sites, when the fire was smoldering and the wind blew directly from the Southwest, the average daily mass of each fraction of ENCF-1 was approximately 2 times higher than that from ENCF-4. Chemical analyses revealed that the coarse PM from both periods contained more Ca^2+^, Mg^2+^, K^+^ and NO_3_^−^ as well as endotoxin levels than the smaller size fractions, which had increased abundance of NH_4_^+^ and SO_4_^2−^. In comparing the two sampling periods, it was clear that the fine and ultrafine PM from ENCF-1 (smoldering stage) had relatively higher organic carbon (OC) content than the ENCF-4 samples (glowing stage); while the coarse PM from ENCF-1 had 70% more LPS than ENCF-4. In contrast, the ENCF-4 fine and ultrafine fractions had approximately double the NH_4_^+^ and SO_4_^2−^ than the ENCF-1 PM suggesting that the PM from this latter period was more characteristic of regional PM pollution. Table 2 shows inorganic elemental constituents from each of the fractions during the two sampling periods. Both ENCF-1 and −4 coarse PM were enriched in crustal elements, such as aluminum [Al], barium [Ba], calcium [Ca], iron [Fe], potassium [K], magnesium [Mg], manganese [Mn], sodium [Na], strontium [Sr] and titanium [Ti], compared to the smaller size fractions, while sulfur [S] content was far higher in the fine and ultrafine fractions. The presence of the high amounts of sodium [Na] in the coarse fractions was possibly related to convective processes from marine sources near the PM sampling location. The overall percent mass balances of the size-fractionated PM samples are shown in Figure 2. As noted above, the ENCF-1 fine and ultrafine PM contained more organic matter than during the ENCF-4 PM sampling period, while the ENCF-4 PM contained more secondary aerosols such as sulfate/ammonium. This is presumably because the smoldering combustion creates large amounts of smoke from the primary source, whereas during the glowing phase very little smoke is emitted and the PM composition is mostly influenced by regional atmospheric chemistry. Between 10-36% of PM remained unidentified, and particularly in the coarse samples, were likely to be insoluble minerals such as aluminum silicates that were resistant to the nitric acid digestion.

**Table 1:** Collected mass, extraction recovery, and chemical compositions in size-fractionated ENCF-1 and −4 PM

		**Collected mass (mg/day)**	**Recovery (%)**	**Ionic components (μg/g)**						**Carbon species**			**Endotoxin (EU/g)**
				**Ca**^**2+**^	**Mg**^**2+**^	**K**^**+**^	**NH**_**4**_^**+**^	**NO**_**3**_^**−**^	**SO**_**4**_^**2−**^	**Total/PM**	**Organic**	**Elemental**	
										**(%)**	**(μg/g)**	**(μg/g)**	
ENCF-1	Coarse	7.8	90	6,162	4,420	4,212	2,983	84,277	29,392	22	217,300	2,300	1,722
	Fine	12.4	93	873	647	2,142	62,648	5,290	225,037	26	254,100	1,650	348
	Ultrafine	3.5	57	2,252	208	2,348	57,045	3,147	187,468	33	325,020	1,500	216
ENCF-4	Coarse	3.6	77	6,117	4,035	5,233	5,403	99,200	36,322	21	210,200	2,650	1,063
	Fine	7.5	101	55	243	1,637	125,133	3,107	564,662	16	155,400	1,000	264
	Ultrafine	1.9	64	0	127	2,010	114,733	1,160	498,458	9	92,340	780	168

**Table 2:** Inorganic elemental constituents of size-fractionated ENCF-1 and -4 PM (μg/g of PM)

		**Al**	**As**	**Ba**	**Ca**	**Cd**	**Cr**	**Cu**	**Fe**	**K**	**Mg**	**Mn**	**Na**	**Pb**	**S**	**Sb**	**Si**	**Sr**	**Ti**	**V**	**Zn**
ENCF-1	Coarse	4,452	38	96	5,926	4	10	38	6,236	5,339	4,827	176	33,303	45	24,378	28	6,580	49	119	20	120
	Fine	2,239	24	67	1,541	4	6	35	2,348	1,430	1,100	67	3,823	51	114,346	16	4,382	28	43	47	195
	Ultrafine	1,417	133	9	850	10	10	63	588	2,139	334	31	1,844	159	150,020	99	4,473	4	26	40	371
ENCF-4	Coarse	3,209	38	103	5,854	3	10	73	4,622	6,177	4,176	122	25,694	45	35,526	28	4,426	39	122	17	348
	Fine	2,213	58	30	671	5	7	54	732	1,305	382	39	2,069	88	310,388	16	1,184	11	22	48	370
	Ultrafine	1,994	133	3	1,310	7	10	99	515	2,131	297	34	2,726	159	390,765	99	13,009	5	41	82	717

### Pulmonary inflammatory responses *in vivo*

**Figure 3:**
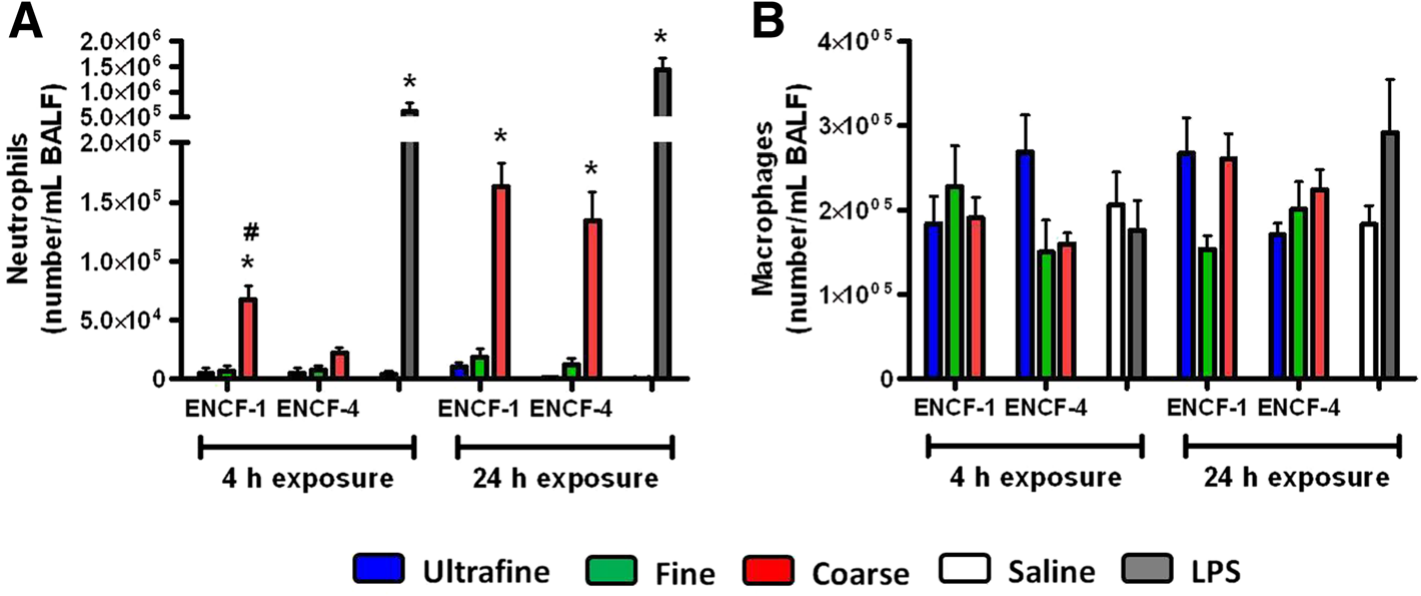
**Number of neutrophils and macrophages in BALF of mice at 4 h and 24 h post-exposure to ENCF-1 and ENCF-4 PM (100 μg) by oropharyngeal aspiration. (A)** neutrophils and **(B)** macrophages. Data are means ± SEM (n = 5–6 in each group). *p < 0.05 compared with the saline-exposed negative control group from the same time point. ^#^p < 0.05 compared with the ENCF-4 PM exposed group from the same time point. Mice exposed to 2 μg of LPS served as a positive control.

**Figure 4:**
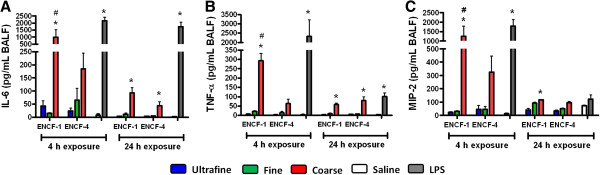
**Cytokine levels in BALF of mice at 4 h and 24 h post-exposure to ENCF-1 and ENCF-4 PM (100 μg) by oropharyngeal aspiration. (A)** IL-6, **(B)** TNF-α, and **(C)** MIP-2 concentrations in BALF. Data are means ± SEM (n = 5–6 in each group). *p < 0.05 compared with the saline-exposed negative control group from the same time point. ^#^p < 0.05 compared with the ENCF-4 PM exposed group from the same time point. Mice exposed to 2 μg of LPS served as a positive control.

**Figure 5:**
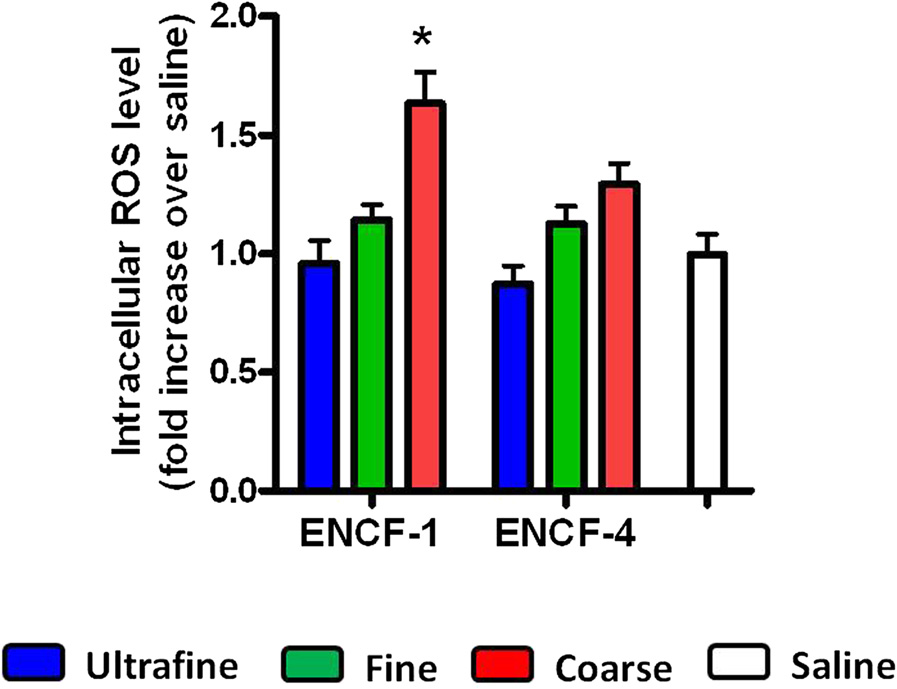
**Intracellular ROS levels in BALF cells of mice at 24 h post-exposure to ENCF-1 and ENCF-4 PM (100 μg) by oropharyngeal aspiration.** Data are means ± SEM (n = 6 in each group). *p < 0.05 compared with the saline-exposed negative control group.

Following exposure to equal masses of the wildfire PM (or LPS as a positive control), biomarkers of lung injury, inflammation and edema in the bronchoalveolar lavage fluid (BALF) of mice were assessed. Lactate dehydrogenase (LDH), N-acetyl-β-D-glucosaminidase (NAG), and γ-glutamyl transferase (GGT) were largely unchanged for any of size-fractionated wildfire PM with the exception that ultrafine PM from ENCF-1 increased LDH at 4 h, and coarse PM from ENCF-4 increased GGT at 24 h (Additional file 1: Figure S1). Concentrations of albumin and total protein from the coarse PM-exposed groups at 24 h were significantly increased compared with saline-exposed groups, indicating increased lung permeability and edema. Only the ENCF-1 coarse PM significantly increased the number of neutrophils at the 4 h time point compared with saline control group (Figure 3A). However, the neutrophil recruitment at 24 h for both the ENCF-1 and -4 coarse samples was significantly increased, accounting for 33 – 35% of the total lavageable cells. As expected, a greater increase in neutrophil numbers followed exposure to the positive control LPS (bacterial endotoxin) at 4 h and 24 h post-exposure, comprising 72 – 76% of the total lavageable cells. None of the PM samples or LPS significantly changed numbers of macrophages in BALF at any time point (Figure 3B). Further analysis of pro-inflammatory cytokines (interleukin-6 (IL-6), tumor necrosis factor-α (TNF-α), and macrophage inhibitory protein-2 (MIP-2)) in BALF (Figure 4) revealed that similar to the neutrophil responses, only ENCF-1 and -4 coarse PM increased these mediators compared to the saline controls and the responses elicited by ENCF-1were significantly higher than ENCF-4. As the levels of all three cytokines diminished by 24 h, the number of neutrophils rose. Exposure to fine and ultrafine PM fractions did not appreciably change cytokine or cell numbers, while LPS caused the largest increases in cytokine and neutrophil levels at both time points. At 24 h we also assessed intracellular reactive oxygen species (ROS) production in the BALF cells (Figure 5). Compared to cells from saline exposed mice, the intensity of oxidized DCF fluorescence (i.e., ROS production) was significantly higher in mice exposed to ENCF-1 coarse PM while no significant differences were noted in any of the other exposure groups. There were no significant changes in circulating white blood cells, red blood cells (RBCs) or RBC indices between the PM-exposed mice and saline controls (data not shown).

### Cardiac toxicity *ex vivo*

**Figure 6:**
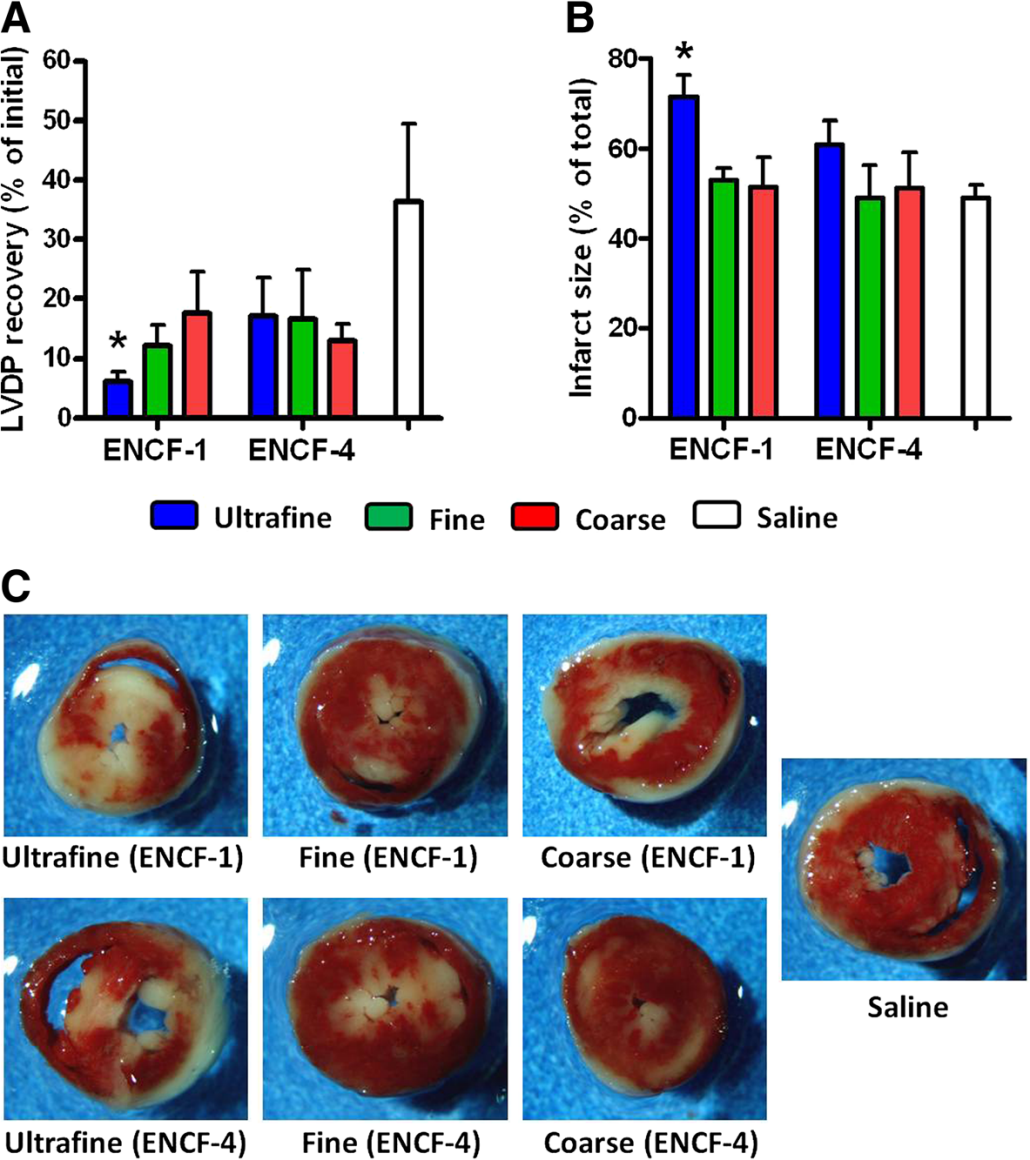
**Post ischemia-reperfusion cardiac end points in mice at 24 h post-exposure to ENCF-1 and ENCF-4 PM (100 μg) by oropharyngeal aspiration. (A)** recovery of LVDP, expressed as a percentage of the initial pre-ischemic LVDP, measured at 1 h of reperfusion after 20 min of ischemia. **(B)** infarct size, expressed as a percentage of the total ventricle area, measured at 2 h of reperfusion after 20 min of ischemia. **(C)** representative images of mouse heart sections stained with TTC. Red-stained areas indicate viable tissue and negatively stained (white) areas indicate infracted tissue. Data are means ± SEM (n = 4–5 in each group). *p < 0.05 compared with the saline-exposed negative control group.

We utilized the Langendorff isolated heart perfusion system to examine the effects of wildfire PM exposure on cardiac function and integrity in mice at 24 h post-exposure. Before initiating ischemia, we measured baseline left ventricular developed pressure (LVDP), heart rate, contractility (e.g., +dP/dt_max_ and -dP/dt_min_) and coronary flow rate. The baseline hemodynamics reported in Table 3 showed no significant change between the different exposure groups and saline controls. LVDP was expressed as the difference in systolic and diastolic pressure in the left ventricle and is the index most often used to monitor cardiac function. Although the hearts from all groups showed a decrease in post-ischemic recovery of LVDP following 20 min of ischemia and 1 h of reperfusion, this effect was only significantly different in mice exposed to the ultrafine particles from ENCF-1 (Figure 6A). Similarly at the end (2 h) of the reperfusion period, only the hearts from ultrafine ENCF-1 exposed mice had significant increases in infarct size as an index of morphological cardiac ischemia-reperfusion damage (Figure 6B). This is further illustrated by representative images from the cross-sectional heart slices stained with 2,3,5-triphenyltetrazolium chloride (TTC), (Figure 6C). Interestingly, mice exposed to ultrafine PM from the later period (ENCF-4) did not show comparable changes in recovery of post-ischemic LVDP or infarct lesion area, indicating that the difference in composition (e.g., the higher organic carbon content in ENCF-1), and not just particle size was responsible for this effect.

**Table 3:** Hemodynamics of perfused hearts* at the end of the control period (before ischemia)

		**n**	**LVDP**	**HR**	**+dP/dt**_**max**_	**-dP/dt**_**min**_	**Coronary flow rate**
			**(cm H**_**2**_**O)**	**(bpm)**	**(cm H**_**2**_**O/sec)**	**(cm H**_**2**_**O/sec)**	**(mL/min)**
ENCF-1	Coarse	5	115 ± 7	357 ± 41	4056 ± 1038	−3271 ± 448	2.2 ± 0.5
	Fine	5	102 ± 21	285 ± 68	3551 ± 750	−2527 ± 758	2.1 ± 0.3
	Ultrafine	5	105 ± 16	345 ± 50	2765 ± 321	−2720 ± 466	1.9 ± 0.4
ENCF-4	Coarse	4	107 ± 19	330 ± 34	3614 ± 572	−2720 ± 923	1.9 ± 0.2
	Fine	4	100 ± 5	306 ± 18	3447 ± 1172	−2665 ± 189	2.1 ± 0.3
	Ultrafine	4	106 ± 10	341 ± 39	3394 ± 481	−2750 ± 414	2.2 ± 0.2
Saline		4	120 ± 16	364 ± 47	4018 ± 1405	−2998 ± 559	2.2 ± 0.3

*Hearts were collected at 24 h post-exposure to ENCF-1 and ENCF-4 PM.

LVDP: left ventricular developed pressure, HR: heart rate, +dP/dt_max_: maximum first derivative of change in left ventricular pressure/time, −dP/dt_min_: minimum first derivative of change in left ventricular pressure/time.

### Pulmonary inflammatory responses *ex vivo*

**Figure 7:**
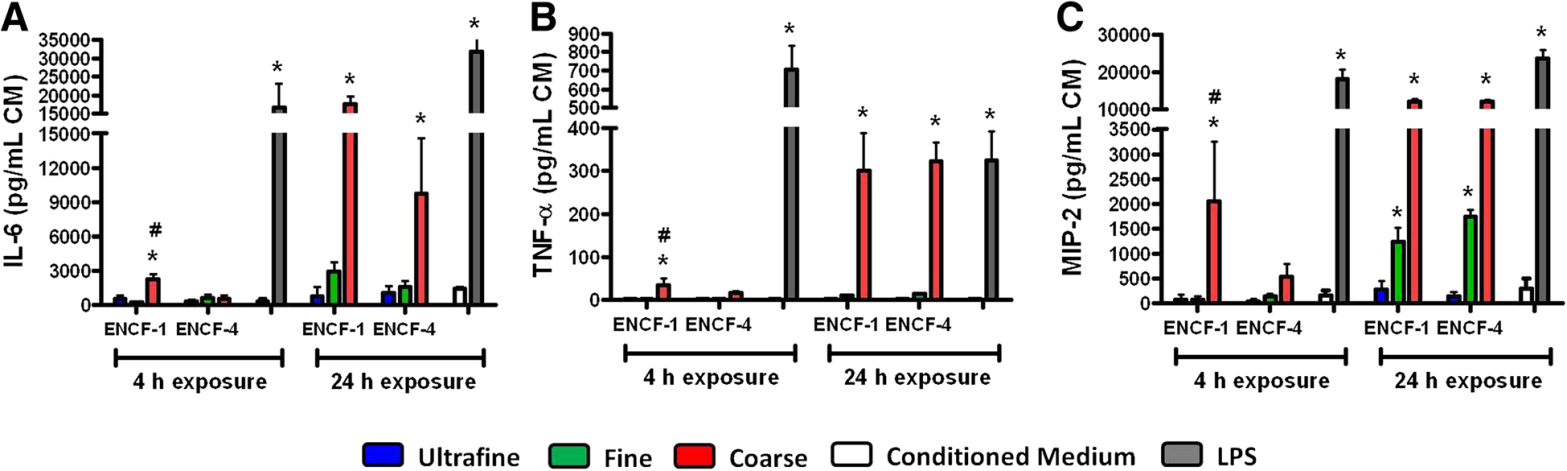
**Cytokine levels in lung tissue slices at 4 h and 24 h post-exposure to ENCF-1 and ENCF-4 PM (11 μg per 8 mm diameter slice). (A)** IL-6, **(B)** TNF-α, and **(C)** MIP-2 concentrations in the conditioned medium (CM) from the lung tissue slices. Data are means ± SEM (n = 3 in each group). *p < 0.05 compared with CM-exposed negative control group from the same time point. ^#^p < 0.05 compared with the ENCF-4 PM exposed group from the same time point. Lung slices exposed to 87 ng/mL of LPS served as a positive control.

Before lung tissue slices were exposed to the wildfire PM, the viability was assessed by both LDH release (an indicator of cell membrane damage) and WST-1 (an indicator of cellular metabolic activity) assays. Quantitative analyses of the released LDH and metabolic activity showed that the lung tissue slices remain viable for up to 6 days in culture (Additional file 2: Figure S2), and based on this, the lung tissue slices were utilized at day 2 of culture. We first monitored biochemical markers for lung injury in the conditioned medium from lung tissue slices at 4 h and 24 h post-exposure to ENCF-1 and -4 PM at 22 μg/mL. In line with the results of lung injury in the *in vivo* study, no significant differences were found in LDH, NAG, and GGT concentrations in the lung tissue slices between the various treatment groups (Additional file 3: Figure S3), indicating that exposure to the wildfire PM in this study did not cause significant cellular damage. To investigate inflammatory responses, we next analyzed production of pro-inflammatory cytokines (i.e., IL-6, TNF-α, and MIP-2) at 4 h and 24 h. Consistent with the *in vivo* results, all three cytokines were increased following exposure to the coarse, but not the fine or ultrafine PM (Figure 7). Moreover, only the ENCF-1 coarse PM significantly increased the levels of all three cytokines at 4 h exposure which was also in accordance with the *in vivo* findings. While we observed a time-dependent decrease in the levels of cytokines *in vivo*, the cytokine levels in the lung tissue slices increased over time with both the PM samples and LPS, possibly because the cytokines accumulated in the conditioned medium in the absence of a circulatory system and other control processes.

**Figure 8:**
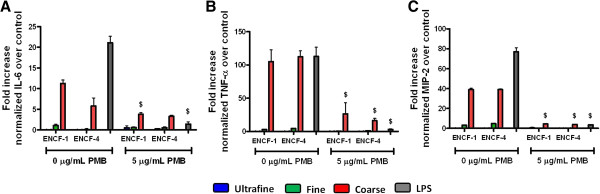
**Inhibition of endotoxin-induced cytokine levels by polymyxin B (PMB) in lung tissue slices at 24 h post-exposure to ENCF-1 and ENCF-4 PM (11 μg per 8 mm diameter slice). (A)** IL-6, **(B)** TNF-α, and **(C)** MIP-2 levels normalized to negative control levels. Data are means ± SEM (n = 3 in each group). ^$^p < 0.05 compared with the PMB non-treated group from the same time point. Lung slices exposed to 87 ng/mL of LPS served as a positive control.

The next set of experiments evaluated the role of endotoxin in PM-induced cytokine production by inhibiting endotoxin with polymyxin B (PMB). First, lung slices were exposed to increasing concentrations (5 – 30 μg/mL) of PMB, indicating that 5 μg/mL PMB resulted in minimal levels of LDH release (Additional file 4: Figure S4). Subsequently lung tissue slices were exposed to 0.5 ml of a suspension containing 22 μg/mL PM pre-treated with 5 μg/mL PMB. At 24 h post-exposure, lung injury biomarkers (LDH, NAG, and GGT) and cytokine levels (IL-6, TNF-α, and MIP-2) were measured in the lung tissue slice supernatant. Again, there were no significant differences in the levels of LDH, NAG, and GGT and this was not affected by PMB treatment (Additional file 5: Figure S5). However, the cytokine responses to the coarse PM from both periods that were pre-treated with PMB were significantly diminished (~56%, ~81%, and ~90% decreases in IL-6, TNF- α, and MIP-2, respectively) compared with those not pre-treated with PMB (Figure 8), indicating that the pro-inflammatory effects were, in large part, attributable to greater LPS content of the coarse PM. LPS that had been pre-treated with PMB also had a 94% decrease in cytokine responses.

## Discussion

Numerous studies of health effects of wildfire smoke exposure have found increased prescription usage, emergency department and hospital admissions for asthma; and increases in bronchitis, pneumonia and cardiovascular symptoms during wildfire events [3,6]. In contrast to forest fires, peat fires produce more smoke, persist for longer periods of time and are notoriously difficult to control [7-9,33]. Little is known about the toxicity of these emissions from different combustion stages however, or if different particle sizes (and resultant chemistry) may influence the effect. Here we investigated pulmonary inflammatory responses and cardiotoxic effects of size-fractionated PM collected from a peat bog wildfire in Eastern North Carolina in the summer of 2008, and also explored the toxicity patterns between *in vivo* and *ex vivo* bioassay systems. The results showed that on an equi-mass basis, the coarse PM collected during the active smolder phase of the wildfire event induced the strongest pro-inflammatory responses in the lung in association with endotoxin content and ROS production, while the ultrafine PM collected from the same period caused significant cardiac effects. In addition, the pulmonary cytokine responses in mice correlated well with those in the lung tissue slice *ex vivo* system.

### Chemical components of wildfire PM affected by wildfire combustion stages

Chemical components of wildfire PM have been investigated from various types of wildfires and during different combustion stages. Compared to emissions from different wildfire types (e.g., savanna, forest, and woodland fires), peat fires produce >100% more CO and >300% more CH_4_, suggesting that they may have a greater impact on human health and climate change [34]. Robinson et al. [35] reported that flaming and smoldering combustion stages of wildfire produced distinctly different ionic and elemental compositions of fine PM, but other size fractions were not assessed. Here we found that the coarse PM from both sampling periods had similar elemental composition, while the fine and ultrafine PM (which form through nucleation, condensation, and coagulation processes) differed between the smoldering and the glowing phases of the burn [36]. In particular, fine and ultrafine PM from ENCF-1 had higher OC content suggesting they were enriched with wildfire smoke, while the PM samples from the ENCF-4 period had higher concentrations of NH_4_^+^ and SO_4_^2−^ indicating that the pollution from this period reflected typical secondary particulate air pollution for the region [37]. The OC mass fractions from ENCF-1 were in good agreement with reported data from the 2005 peat fires in Indonesia [38], while forest fires often produce even higher OC levels (typically > 90% by mass) [35], suggesting that OC levels are affected not only by the different combustion stages but also the type (i.e., fuel source) of wildfires. It should be noted that the wind speed during ENCF-1 was higher than during ENCF-4 and the prevailing wind was from the South-west, while ENCF-4 wind conditions were in general lighter and more southerly in nature with some periods of wind from other directions. Despite this caveat, it is clear from the up- and down-wind regional monitors, that the ENCF-1 samples were enriched for wildfire-type pollutants and provided the contrast with ENCF-4 that we intended to explore in this study.

### Acute lung inflammation caused by coarse but not smaller size fractions

We and others have previously shown that coarse PM from a variety of different urban locations causes much more lung inflammation than fine or ultrafine fractions, and these effects have been attributed to numerous components including transition metals, crustal material as well as bioactive components such as LPS [39-41]. Similarly we found that on an equivalent mass basis (100 μg PM exposure), the wildfire coarse PM caused relatively more pulmonary toxicity than coarse PM collected from either traffic- or urban derived PM from North Carolina [30,32], and the effect was at least, in part, explained by endotoxin levels. Studies from other wildfire events have similarly found that coarse PM derived from wildfires induce larger lung inflammatory responses compared with smaller-sized fractions [13]. Whether the LPS originated from the smoke itself or was associated with differences in meteorological conditions of agricultural practices that varied across the two periods is difficult to discern. While it is clear that coarse PM collected from agricultural areas contain more LPS and is associated with increased toxic and pro-inflammatory effects [42], it is also known that cigarette smoke which is another example of smoldering combustion of agricultural products also contains significant levels of bacterial endotoxin and may, contribute to smoking-related pulmonary diseases [16]. Furthermore, while forest fires (e.g., California wildfires) are dominated by flaming and higher temperature, which would normally degrade LPS [7,16], peat fires normally burn at lower temperature and can be pyrolyzed between 200-250°C. It is also worth noting that peat is made up of organic materials partially decomposed by bacteria, fungi, insects, and worms, and when burned, produces massive amount of organic aerosol including endotoxins [17,43]. While the chemical composition of wildfire PM is variable across forest, grassland, agricultural residue, and peat fires [44,45], polycyclic aromatic hydrocarbons (PAH) are relatively low in abundance in most wildfire-derived PM, and particularly in relation to emissions from peat fires [14,38,46].

In addition to the significant association of endotoxin with pulmonary inflammation, we also demonstrated that the observed pulmonary inflammatory effect of wildfire coarse PM increased intracellular ROS production in BALF cells. Although we did not determine which chemicals of the wildfire PM in this study were responsible to induce oxidative stress, the coarse fraction of wildfire PM has been reported to have higher concentrations of carbon-centered radicals that are capable of inducing redox cycling in cells, leading to high oxidative stress [14,47,48]. Although both ENCF-1 and −4 coarse PM had a similar organic component fraction (~45%) the ENCF-1 sample induced higher intracellular ROS production suggesting that it had different chemistry and higher concentrations of the carbon-radicals than ENCF-4 coarse PM. In addition to the size-dependent pro-inflammatory responses of wildfire PM, production of pro-inflammatory cytokines and activation of neutrophil recruitment occurred in a time-dependent manner. Airway (or alveolar) epithelial cells, macrophages, fibroblasts, and/or endothelial cells rapidly produce cytokines (e.g., IL-6 and TNF-α) and chemokines (e.g., MIP-2) after exposure to ambient PM which gradually diminishes as inflammatory cell number increases [42,49-52]. Often macrophage numbers are initially decreased due to their tighter adhesion to the alveolar epithelium and an increased numbers are only seen later in demand for enhanced particle clearance [53]. Here we found no significant changes in macrophage numbers up to 24 h post-exposure, but did observe a pronounced early cytokine response followed by a later influx of neutrophils which is consistent with other findings in studies on wildfire PM exposures [14,15,54]. Swiston et al. [54] reported increased levels of circulating cytokines (e.g., IL-6), and neutrophils (but not macrophages) in the sputum of healthy fire fighters after a day of fire-fighting. In an *in vivo* study on the toxicology of wildfire PM, Wegesser et al. [14] demonstrated that concentrations of inflammatory cytokines/chemokines (e.g., TNF-α) in BALF from mice were significantly increased at 6 h post-exposure to wildfire coarse and fine PM, followed by a sharp decrease at 24 h. Exposure of a mouse macrophage cell line (RAW 264.7) to wildfire PM at 24 h increased inflammatory cytokine responses (IL-6, TNF-α and MIP-2) in a dose-dependent manner, and the coarse PM was the most potent stimulator for the cytokine responses [13].

### Cardiac dysfunction caused by ultrafine PM collected during the active wildfire

In addition to the pulmonary effects, cardiac function and injury induced by the wildfire PM were investigated at 24 h post-exposure. Ultrafine PM collected during smoldering stage (ENCF-1) but not glowing stage (ENCF-4), caused significant adverse cardiac effects (e.g., low recovery of post-ischemic LVDP and increased infarct size). Since this fraction had approximately three-fold greater organic matter content than ENCF-4, these adverse effects were likely due to source dependent differences in chemistry. In a similar vein, we have previously reported that near road, but not far road, ultrafine PM had similar effects in this same cardiac reperfusion model compared with the saline control group [30]. Taken together, the results suggest that the size (i.e., ultrafine) as well as chemical composition of these particles influences cardiac toxicity. Although more studies are needed to understand the mechanism(s) of this effect, it is clear that cardiovascular function can be altered by various chemical components including organic compounds, trace metal oxides, and elemental carbon [55]. It should be noted that several epidemiological studies report no significant association between cardiovascular effects and smoke exposure from forest wildfires [56,57] which may possibly be explained by the difference in chemistry between forest and peat wildfire smoke. On the other hand, peat fires have been associated with increased ED visits for heart failure [29], consistent with the cardiac effects of ENCF-1 ultrafine PM presented here.

### *Ex vivo* lung tissue slices as a good surrogate for *in vivo* toxicity screening and testing

The aims of this study were not only to assess toxicity of wildfire PM *in vivo* but also to investigate whether the *in vivo* effects could be predicted by *ex vivo* models (i.e., lung tissue slices). For the past 25 years, lung tissue slices have been successfully developed and applied in the research fields of physiology, pharmacology, pathology, and toxicology [23-28], however few studies have compared response patterns between *in vivo* and *ex vivo* models [58]. In this respect, a promising finding was that the lung tissue slice model showed a similar pattern of responses to those seen in mice. Additionally, the lung tissue slices demonstrated very low baseline cytokine production but readily responded to bioactive PM samples and LPS. Exposure to wildfire PM induced differential cytokine responses in BALF of mice characterized by 3–5 times greater responses of cytokine IL-6 and MIP-2 than those of TNF-α and a similar differential cytokine response was observed in lung tissue slices. Besides showing concordance with these three cytokines, we also found that IL-1β was not increased in either the *in vivo* and *ex vivo* systems (data not shown), suggesting that the lung slices produced similar responses to the whole lung environment. Due to the fact that there are more than 40 different cell types in the mammalian lung [59], *in vitro* cell culture models of one or multiple cell types present challenges in terms of interpretation of cellular toxicity, physiology, and morphology in relation to complex cell-cell contacts and cell-matrix interactions *in vivo*. For example, it has been reported that slightly different genotoxic responses to wood smoke were observed in alveolar epithelial cells and monocytes [60]. Furthermore, human alveolar epithelial type 2 cell line (A549) and primary rat type 2 cells showed different inflammatory responses to ambient PM exposures [61]. From a practical point of view, it is relatively straightforward to prepare lung tissue slices and also easy to maintain high cell viability in culture, compared to primary cells or cell lines. In addition to the structural and morphological similarities to the whole organ, application of this technique greatly reduces the number of animals needed. In this study, we were able to generate more than 10 lung tissue slices from one mouse lung, thus requiring approximately 10-fold less number of animals. Ideally mouse lung tissue should be replaced possible by human cells and tissues to avoid species differences that complicate risk assessment, not only from a dosimetric perspective, but because of differences in biochemistry and (patho) physiological responses [62]. Since there are many issues (e.g., ethical, legal, logistical, safety, and quality) relating to the use of human tissue for research [63] however, normal human lung tissue is not widely available for use in laboratories across the world.

The advantages of the lung tissue slice technique lead us to investigate the contribution of endotoxin to the observed inflammatory effect of wildfire PM in this study. The lung slices effectively responded to endotoxin and the coarse PM and this was significantly inhibited by PMB. Since the inflammatory responses to the particles were not completely inhibited by PMB other microbial components may also have played a role [64] or the binding of PMB may have been reduced by the presence of hydrophobic molecules [65]. Overall however, the results show that the lung tissue slices could be used as a surrogate for *in vivo* toxicity screening and testing to identify and discriminate key factor(s) that induce pulmonary toxicity.

## Conclusions

We conclude that with equal mass exposure, pulmonary toxicity of wildfire PM was limited to the coarse fraction which induced substantial cytokine (IL-6, TNF-α, and MIP-2) release, neutrophil recruitment, and increased protein into the mouse lungs, while the ultrafine fraction collected during the active part of the wildfire had only cardiac effects. Chemical analysis revealed that PM obtained during the wildfire had relatively higher organic matter content, and the coarse PM had markedly higher endotoxin content compared to fine and ultrafine PM. Production of ROS in BALF cells was significantly increased by the ENCF-1 coarse PM and was likely associated with both the increase in cytokines and the deep lung permeability (i.e., edema) noted *in vivo*. Furthermore, the lung slice responses were reduced by inhibiting endotoxin in the coarse PM. Overall, the lung tissue slice system showed similar pro-inflammatory responses to those seen *in vivo* and provides a predictive, reliable, and reproducible alternative assay system for acute pulmonary toxicity testing. The purpose of the study was to provide a mass-based comparison of the different PM materials as opposed to generating dose response information on individual samples. It should be noted however; that PM concentrations occurring during wildfires would be incrementally higher than during other times, therefore the responses to equivalent masses of PM (as shown here) likely underestimated the effect of a temporal change in the amount and type of particulate air pollution. Finally, knowledge of the causal components of the wildfire smoke and mechanisms of effect could aid in the specificity of regional public health alerts, and may also provide strategies for chemo-prevention in first responders and the population at risk.

## Materials and methods

### Experimental animals

Adult pathogen-free female CD-1 mice (~20-25 g and ~30-45 g, body weights for pulmonary toxicity and cardiac toxicity and lung tissue slice studies, respectively) purchased from Charles River Breeding Laboratories (Raleigh, NC). Younger mice (8–10 weeks) were selected for the aspiration studies, while the larger, older mice (16–20 weeks) were used for the heart perfusion and lung slice preparations. Mice were housed in groups of five in polycarbonate cages with hardwood chip bedding at the U.S. Environmental Protection Agency (EPA) Animal Care Facility accredited by the Association for Assessment and Accreditation of Laboratory Animal Care and were maintained on a 12-hour light to dark cycle at 22.3 ± 1.1°C temperature and 50 ± 10% humidity. Mice were given access to rodent chow and water ad libitum and were acclimated for at least 10 days before the study began. The studies were conducted after approval by the EPA Institutional Animal Care and Welfare Committee.

### Eastern North Carolina Wildfire (ENCF) PM collection

Wildfire-generated PM samples were collected at a location in Camden County, NC, approximately 40 miles north-northeast of a peat-bog wildfire at the Pocosin Lakes National Wildlife Refuge in Eastern North Carolina. The wildfire started on 06/01/2008 and was 100% contained on 09/24/2008. The ENCF-1 PM sample was collected from 06/26/2008-07/11/2008 when the fire was still smoldering, and the ENCF-4 PM sample was collected from 08/09/2008-08/19/2008 after the fire had been controlled but not fully extinguished. PM samples were collected using a ChemVol high-volume cascade impactor (model 2400, Rupprecht & Patashnick Co., Albany, NY), and were size-fractionated in three different ranges (ultrafine: < 0.1 μm; fine: 0.1-2.5 μm; coarse: 2.5-10 μm). The coarse and fine PM and the ultrafine PM were collected onto precleaned/preweighted polyurethane foam (PUF) and polyproplylene fiber filter (PPF), respectively. The amount of PM collected was determined gravimetrically.

### Wildfire PM extraction and recovery

The size-fractionated PM was extracted for chemical and toxicological analyses. PUF (for coarse and fine PM) and PPF (for ultrafine PM) substrates were split into thirds and transferred to sterile glass 15 mL and 50 mL tubes and fully submerged in 13 mL and 30 mL of analytical grade methanol (Sigma, St. Louis, MO), respectively. The tubes were placed in an ultrasonic water bath at < 28°C for 1 h at 100% power, 25 kHz frequency, and sweep ultrasonic mode (model TI-H15, Elma Hans Schmidbauer GmbH & Co. KG, Singen, Germany). After sonication, a sterile polypropylene rod was pressed against the PUF and PPF to help dislodge PM from the foam substrates. Extracted PM suspensions in methanol were then decanted into pre-weighed sterile polypropylene 15 mL (for coarse and fine PM) and 50 mL (for ultrafine PM) tubes, respectively. In order to remove any loose polypropylene fibers dislodged during extraction of the ultrafine fractions, the PM suspension was poured through a 70 μm pore filter. Aliquots of all size-fractionated PM suspensions obtained from one third of the entire filter were evaporated to dryness under nitrogen and the tubes were reweighed. PM recovery efficiency was calculated as the difference between the pre- and post-weight of the tubes, divided by one third of the PM mass collected on the filters. After determination of PM mass in the tube, the dried PM of each sample was dissolved in 100% methanol and an aliquot removed for chemical analysis. The remaining sample was brought up with 0.9% sterile saline to produce a 10 mg/mL PM suspension containing 0.5% methanol. The reconstituted PM suspensions and 0.5% methanol in saline were sonicated, vortexed for 1 min, and 1 mL aliquots of the PM suspensions were stored at −80°C until toxicity testing.

### Wildfire PM chemical analysis

Each PM suspension was vortexed and sonicated to ensure homogeneity, then aliquoted for chemical analysis of elemental, ionic, and carbon fraction content. Briefly, for inorganic elemental analysis, 2-mg aliquots of PM suspension in water were digested in 10% nitric acid at 60°C for 4 h to solubilize the metals and supernatants separated by centrifugation were diluted to a final concentration of 1.5% nitric acid, then assayed for 27 inorganic elements by inductively coupled plasma-optical emission spectrometry (ICP-OES, using the U.S. EPA Method 200.7 rev4.4 protocol [66]). For ionic component analysis, 300-μg aliquots of PM suspension in water were diluted in 10 mL of ultrapure water and were analyzed for nitrate (NO_3_^−^), sulfate (SO_4_^2−^), chloride (Cl^−^), sodium (Na^+^), ammonium (NH_4_^+^), potassium (K^+^), magnesium (Mg^2+^) and calcium (Ca^2+^) by ion chromatography (IC; using U.S. EPA Compendium Method 10–4.2 [67]). For carbon species analysis, 300-μg aliquots of PM suspension in water were pipetted onto pre-baked 1.5 cm^2^ quartz filters, dried, and analyzed by a thermo-optical method based upon sequential pyrolytic vaporization and detection by transmittance using a carbon analyzer (model 107A; Sunset Laboratory Inc., Tigard, OR). In order to estimate total mass of organic compounds in wildfire PM, we multiplied a measured organic carbon mass by the mass ratio of organic matter (OM) to organic carbon (OC). For wildfire or biomass burning PM, the estimate of OM/OC ratio from the literature is approximately 2.1 [35,68]. A suspension of standard reference material (ambient urban atmospheric particulate NIST 1649a) was prepared in the same concentration as the study PM samples and used to track recovery of major elements, ions, and carbon content.

### Endotoxin measurement

Each PM suspension was vortexed and sonicated to ensure homogeneity, and then diluted in endotoxin-free water at a concentration of 1 mg/mL, followed by centrifugation for 10 min at 10,000×g. Supernatants were collected to determine endotoxin concentrations in PM suspensions. Endotoxin measurements were performed using the Limulus amebocyte lysate assay (QCL-1000; Lonza, Walkersville, MD) as per the manufacturer’s protocol.

### *In vivo* toxicity of wildfire PM

#### Mouse exposure to wildfire PM

Oropharyngeal aspiration was performed on mice anesthetized in a small plexiglass box using vaporized anesthetic isofluorane, following a technique described previously [31]. Briefly, the tongue of the mouse was extended with forceps and 100 μg of PM in 50 μL saline was pipetted into the oropharynx. Immediately, the nose of the mouse was then covered causing the liquid to be aspirated into the lungs. Similarly, a separate group of mice was instilled with 2 μg of lipopolysaccharide (LPS; Escherichia coli endotoxin; 011:B4 containing 10^6^ unit/mg material; Sigma) as a positive control to demonstrate maximal responsiveness to this well characterized inflammatory agent. Additional mice were instilled with 50 μL saline alone as a negative control. The selection of dose was based on the following information. It has been reported that PM_3.5_ or PM_10_ concentrations near wildfires are as high as ~2000 – 2800 μg/m^3^[3,54]. Therefore, wildfire PM deposited in the human lungs for 24 h in this particular case (assuming a human respiratory volume of 28,800 L/day and ~70 m^2^ surface area of airspaces of the lung) would be ~82.2 – 115.2 ng/cm^2^. Moreover, assuming a mouse respiratory volume of 38.7 L/day [69], mice could inhale between 77 and 108 μg of wildfire PM over a 24 h period. Since we intended to evaluate acute lung toxicity of wildfire PM that represents a peak 24 h exposure for a wildfire event, the single PM dose (100 μg) was chosen in this study and also the PM dose (equivalent to 154 ng/cm^2^ in mouse lungs: see below for a more detailed calculation) appeared to be relevant to the inhaled wildfire PM concentrations in the human lungs.

#### Bronchoalveolar lavage and hematology

At 4 h and 24 h post-exposure, 6 mice from each treatment group were euthanized with 0.1 mL intraperitoneal injection of Euthasol (diluted 1:10 in saline; 390 mg pentobarbital sodium and 50 mg phenytoin/mL; Virbac AH, Inc., Fort Worth, TX), and blood was collected by cardiac puncture using a 1-mL syringe containing 25 μL sodium citrate to prevent coagulation. The trachea was then exposed, cannulated and secured with suture thread. The thorax was opened and the left mainstem bronchus was isolated and clamped with a microhemostat. The right lung lobes were lavaged three times with a single volume of warmed Hanks balanced salt solution (HBSS; 35 mL/kg mouse). The recovered bronchoalveolar lavage fluid (BALF) was centrifuged at 800×g for 10 min at 4°C and the supernatant was stored at both 4°C (for biochemical analysis) and −80°C (for cytokine analysis). The pelleted cells were resuspended in 1 mL HBSS (Sigma). Total BALF cell count of each mouse was obtained by a Coulter counter (Coulter Co., Miami, FL). Additionally, 200 μL resuspended cells were centrifuged in duplicate onto slides using a Cytospin (Shandon, Pittsburgh, PA) and subsequently stained with Diff-Quik solution (American Scientific Products, McGraw Park, PA) for enumeration of macrophages and neutrophils with at least 200 cells counted from each slide. Hematology values including total white blood cells, total red blood cells, hemoglobin, hematocrit, mean corpuscular volume, mean corpuscular hemoglobin concentration, and platelets were measured using a Coulter AcT 10 Hematology Analyzer (Beckman Coulter Inc., Miami, FL).

#### Biochemical analysis

Concentrations of lactate dehydrogenase (LDH) and γ-glutamyl transferase (GGT) were determined in BALF using commercially available kits (Thermo Scientific, Middletown, VA). Albumin and total protein concentrations were measured by the SPQ test system (DiaSorin, Stillwater, MN) and the Coomassie plus protein assay (Pierce Chemical, Rockford, IL) with a standard curve prepared with bovine serum albumin (Sigma), respectively. Activity of N-acetyl-β-D-glucoaminidase (NAG) was determined using a NAG assay kit (Roche Applied Science, Indianapolis, IN). All assays were modified for use on the KONELAB 30 clinical chemistry spectrophotometer analyzer (Thermo Clinical Lab Systems, Espoo, Finland) as described previously [31].

#### Cytokine analysis

Concentrations of tumor necrosis factor-α (TNF-α), interleukin-1β (IL-1β), interleukin-6 (IL-6) and macrophage inhibitory protein-2 (MIP-2) in BALF were determined using commercial multiplexed fluorescent bead-based immunoassays (Milliplex Map Kit, Millpore Co., Billerica, MA) measured by a Luminex 100 (Luminex Co., Austin, TX) following the manufacturer’s protocol. The limits of detection (LOD) of each cytokine were 6.27, 14.6, 3.28 and 29.14 pg/mL for TNF-α, IL-1β, IL-6 and MIP-2, respectively, and all values below these lowest values were replaced with a fixed value of one-half of the LOD value.

#### Intracellular reactive oxygen species (ROS) analysis

BALF cells were also used to determine whether ROS production was induced by the wildfire PM exposure. We used nonfluorescent 2’ , 7’-dichloro-fluorescein diacetate (H_2_DCFDA, Invitrogen, Carlsbad, CA) to measure the intracellular ROS production because H_2_DCFDA easily diffuses into cells and is converted to highly fluorescent DCF by intracellular ROS. At 24 h post-exposure, BALF cells were assessed by plating 10,000 cells onto cell culture plates and incubating the cells with H_2_DCFDA at 10 μM for 2 h. Fluorescence of the probe was quantified using a fluorescence plate reader (SpectraMax Plus 384, Molecular Devices, Sunnyvale, CA), with excitation at 485 nm and emission at 530 nm.

#### Cardiac function and necrosis evaluation

At 24 h post-exposure, hearts were isolated for cardiac perfusion as described previously [32]. Briefly, hearts from the PM- and saline-exposed mice were perfused for 25 min prior to initiating 20 min of global no-flow ischemia followed by 2 h of reperfusion. Recovery of post-ischemic left ventricular developed pressure (LVDP), expressed as a percentage of the initial pre-ischemic LVDP was measured at 1 h of reperfusion by a PowerLab system (AD Instruments, Milford, MA). In addition, heart rate and contractility (dP/dt) data were continuously collected. At 2 h of reperfusion, the hearts were stained with 2,3,5-triphenyltetrazolium chloride (TTC; Sigma) and then fixed in formalin to evaluate cardiac necrosis (i.e., infarct size measurement). The area of necrosis was measured by taking cross-sectional slices. Infarct size was quantified by measuring the areas of stained versus unstained tissue and expressed as a percentage of the total ventricular section with the use of Adobe Photoshop.

### *Ex vivo* toxicity of wildfire PM

#### Mouse lung slice preparation and incubation

Lung slices were prepared as previously described with some modifications [23,25,70,71]. Briefly, mice were euthanized with 0.1 mL intraperitoneal injection of Euthasol (diluted 1:10 in saline; Virbac AH, Inc.) and exsanguinated by cutting the vena cava. The trachea was exposed and cannulated using a 20G luer stub adapter (Instech Solomon, Plymouth Meeting, PA). The lungs were filled with 1.5% (w/v) low-melting agarose (Sigma) in minimum essential medium (MEM; Sigma) at 37°C (~44 mL/kg mouse) for ~1 min, followed by injecting ~0.1 mL of air to flush the agarose-MEM out of the airways into alveolar tissue. The lungs were rinsed with the ice-cold slicing buffer solution (Earle’s balanced salt solution (Sigma) supplemented with 15 mM N-(2-hydroxyethyl) piperazine-N’-(2-ethanesulfonic acid) hemisodium salt (HEPES; Sigma)) and removed from the mouse. The lungs were transferred into the ice-cold slicing buffer solution to further solidify the agarose and then the lung lobes were separated using a surgical blade, and the lung tissue cores (8 mm diameter) were prepared using a tissue coring tool (Alabama Research and Development, Munford, AL). Tissue cores were cut into 350 μm thick slices in the ice-cold slicing buffer solution using a specialized vibratome (OTS 5000, FHC Inc., Bowdoinham, ME). The lung tissue slices were then incubated in the wash buffer solution (Dulbecco’s modified eagle’s medium/nutrient mixture F-12 Ham (Sigma) supplemented with 100 units/mL penicillin (Sigma) and 100 μg/mL streptomycin (Sigma)) at 37°C, 5% CO_2_ and 95% air humidity under cell culture conditions for 4 h. The wash buffer solution was changed every 30 min for 2 h, followed by changing the solution every hour for the next 2 h to remove the re-solubilized agarose. The lung tissue slices were then transferred into a tissue culture treated polystyrene 48-well plate (Corning Inc., Corning, NY) and cultured in the slice incubation medium (the wash buffer solution supplemented with 200 mM L-glutamine (Sigma), 0.1 mM MEM non-essential amino acids (Sigma) and 15 mM HEPES) for up to 6 days at 37°C in a humidified atmosphere of 5% CO_2_ and 95% air. The lung tissue slices received fresh media every day.

#### Mouse lung slice viability assessments

Viability of mouse lung slices was tested by measuring leakage of LDH from the lung slices using an LDH assay kit (Roche Applied Science) and enzymatic activity based on the cellular cleavage of water-soluble tetrazolium salt (WST-1) to formazan in the lung slices using a WST-1 assay kit (Roche Applied Science) for up to 6 days in culture. Released LDH and soluble formazan in supernatant of mouse lung slice culture medium were quantified by a spectrophotometer (SpectraMax Plus 384). Mouse lung slices exposed to 0.3% Triton X-100 for 15 min at 37°C served as a positive control.

#### Mouse lung slice exposure to wildfire PM

Reconstituted PM suspensions were sonicated for 2 min, vortexed for 1 min and further diluted with the slice incubation medium to achieve a concentration of 22 μg/mL. On day 2 of culture, lung slices were exposed to PM at 22 μg/mL for 4 h and 24 h, respectively. The concentration of 22 μg/mL (total volume of 0.5 mL, therefore 11 μg of PM per slice) was estimated to be five times higher than the *in vivo* exposure dose used in this study. If it is assumed that the lung surface area of a 20 g mouse is ~650 cm^2^, 1 cm^3^ mouse lung tissue has ~800 cm^2^ lung surface area, and 100% of oropharyngeal instilled PM is delivered to the lungs, 100 μg of PM dose in a mouse (~650 cm^2^ lung surface area) is equivalent to 2.2 μg of PM dose in a mouse lung slice (~14 cm^2^ lung slice surface area) [72]. Mouse lung slices were exposed to 87 ng/mL LPS which was an equivalent concentration *in vivo* and served as a positive control. Mouse lung slices exposed to the conditioned medium alone served as a negative control. At 4 h and 24 h post-exposure, lung slice culture fluids were collected, centrifuged at 10,000×g for 5 min, and culture supernatants were stored at both 4°C (for extracellular biochemical analysis) and −80°C (for cytokine analysis). Subsequently, mouse lung slices were homogenized using a tissue homogenizer in a lysis buffer solution containing 0.5% Triton X-100, 150 mM NaCl, 15 mM Tris–HCl (pH 7.4), 1 mM CaCl_2_ and 1 mM MgCl_2_[73]. Homogenates were then centrifuged at 10,000×g for 10 min and supernatants were stored at −80°C (for intracellular biochemical analysis, e.g., GGT).

#### Endotoxin inhibition by polymyxin B (PMB) treatment

PMB is an antibiotic primarily used for inhibition of almost all gram-negative bacteria at low concentrations, while PMB also induces damage to cytoplasmic membranes at relatively high concentrations [74]. Lung tissue slices were exposed to different concentrations of PMB (5–30 μg/mL) for 24 h to determine the concentration at which PMB toxicity occurs. The concentration of 5 μg/mL PMB was found to have a minimal toxic effect in the lung tissue slices. Each PM suspension was then treated with 5 μg/mL PMB at room temperature for 30 min. Subsequently, lung tissue slices were exposed to the PM (or LPS) pre-treated with PMB for 24 h. LPS pre-treated with 5 μg/mL PMB was used as a positive control. At 24 h post-exposure, lung slice culture fluids were collected, followed by centrifugation, and culture supernatants were stored at both 4°C (for extracellular biochemical analysis) and −80°C (for cytokine analysis). Supernatants of tissue homogenates were also collected and stored at −80°C (for intracellular biochemical analysis).

#### Biochemical and cytokine analysis

Similar to the *in vivo* lung inflammation analyses described above, the supernatants of tissue culture fluids and tissue homogenates after exposure to PM (or PM pre-treated with PMB) were used to determine the extracellular (LDH and NAG) and intracellular (GGT) biochemical analyses as well as cytokine analysis (IL-1β, IL-6, TNF-α, and MIP-2). Biochemical analyses were performed using a KONELAB 30 clinical chemistry spectrophotometer analyzer (Thermo Clinical Lab Systems). Cytokine analysis was performed using multiplexed fluorescent bead-based immunoassays (Milliplex Map Kit) measured by the Luminex 100 (Luminex Co).

### Statistical analysis

Data were expressed as means ± the standard error of the mean (SEM). The results of the PM-exposed groups were compared to those of the negative control group (*in vivo* and *ex vivo*) or those of the PM with PMB-exposed groups (*ex vivo*). Statistical comparison of the PM-exposed groups to the negative control group was performed by one-way analysis of variance (ANOVA) followed by the Dunnett’s multiple comparison post-hoc test, otherwise significance for 3 ≥ PM-exposed groups was determined by one-way ANOVA, as appropriate, with the Newman-Keuls post-hoc test. Statistical analyses were performed using commercial software (GraphPad Prism 4.03, GraphPad Software, Inc., San Diego, CA). If the data did not meet the ANOVA assumptions of either normality or equal variances (Levene’s test; p > 0.05), the data were transformed. Subsequent to the transformation, the data were checked for requirement compliance and if acceptable, ANOVA proceeded. The statistical significance level was assigned at a probability value of p < 0.05.

## Abbreviations

BALF: Bronchoalveolar lavage fluid; CM: Conditioned medium; ED: Emergency department; ENCF: Eastern North Carolina wildfire; GGT: γ-glutamyl transferase; HBSS: Hanks balanced salt solution; HEPES: N-(2-hydroxyethyl) piperazine-N’-(2-ethanesulfonic acid) hemisodium salt; H_2_DCFDA: 2’,7’-dichloro-fluorescein diacetate; IL-1β: Interleukin-1β; IL-6: Interleukin-6; LDH: Lactate dehydrogenase; LPS: Lipopolysaccharide; LVDP: Left ventricular developed pressure; MEM: Minimum essential medium; MIP-2: Macrophage inhibitory protein-2; NAG: N-acetyl-β-D-glucoaminidase; OC: Organic carbon; OM: Organic matter; PAH: Polycyclic aromatic hydrocarbons; PBS: Phosphate buffered saline; PM: Particulate matter; PMB: Polymyxin B; PPF: Polypropylene fiber filter; PUF: Polyurethane foam; ROS: Reactive oxygen species; TNF-α: Tumor necrosis factor-α; TTC: 2,3,5-triphenyltetrazolium chloride; WST-1: Water-soluble tetrazolium salt.

## Competing interests

The authors declare that they have no conflict of interests.

## Author’s contributions

YHK contributed to the experimental design, carried out the pulmonary assessment, cardiac evaluation, and lung tissue slice experiment, performed the data analyses and figure generations, and drafted the manuscript. HT performed the cardiac evaluation. MD and EB performed the pulmonary assessment and helped with data analyses. QTK collected the wildfire PM samples. JM, KK and MH characterized the wildfire PM samples. JAD performed ROS assessments, assisted with data interpretation and manuscript preparation. MIG conceived and designed the experiment, evaluated the results, and co-wrote the manuscript. All of the authors read and approved the final manuscript.

## Supplementary Material

Additional file 1: Figure S1 Biochemical markers for lung injury and edema in BALF of mice at 4 h and 24 h post-exposure to ENCF-1 and ENCF-4 PM (100 μg) by oropharyngeal aspiration. **(A)** LDH, **(B)** NAG, **(C)** GGT, **(D)** albumin, and **(E)** total protein concentrations in BALF. Data are means ± SEM (n = 5–6 in each group). *p < 0.05 compared with the saline-exposed negative control group from the same time point. Mice exposed to 2 μg of LPS served as a positive control. Click here for file

Additional file 2: Figure S2 Viability of lung tissue slices during 6 days of culture. **(A)** LDH assay and **(B)** WST-1 assay. Data are means ± SEM (n > 3 in each group). Lung slices exposed to 0.3% Tritron X-100 for 15 min served as a positive control. Click here for file

Additional file 3: Figure S3 Biochemical markers for lung injury in lung tissue slices at 4 h and 24 h post-exposure to ENCF-1 and ENCF-4 PM (22 μg/mL). **(A)** LDH, **(B)** NAG, and **(C)** GGT concentrations in the conditioned medium (CM) from the lung tissue slices. Data are means ± SEM (n = 3 in each group). Lung slices exposed to 87 ng/mL of LPS served as a positive control. Click here for file

Additional file 4: Figure S4 Dose-dependent LDH release in lung tissue slices exposed to polymyxin B at 24 h. Data are means ± SEM (n > 3 in each group). Lung slices exposed to 0.3% Tritron X-100 for 15 min served as a positive control. Click here for file

Additional file 5: Figure S5 Biochemical markers for lung injury in lung tissue slices at 24 h post-exposed to ENCF-1 and ENCF-4 PM (22 μg/mL) with and without pre-treatment of polymyxin B (PMB). **(A)** LDH, **(B)** NAG, and **(C)** GGT concentrations in the conditioned medium (CM) from the lung tissue slices. Data are means ± SEM (n = 3 in each group). Lung slices exposed to 87 ng/mL of LPS served as a positive control. Click here for file
