# Supplementary figures and images for: Cardiopulmonary toxicity of peat wildfire particulate matter and the predictive utility of precision cut lung slices
Source: Part Fibre Toxicol. 2014 Jun 16;11:29. doi: 10.1186/1743-8977-11-29 (PMC4072480; doi:10.1186/1743-8977-11-29)

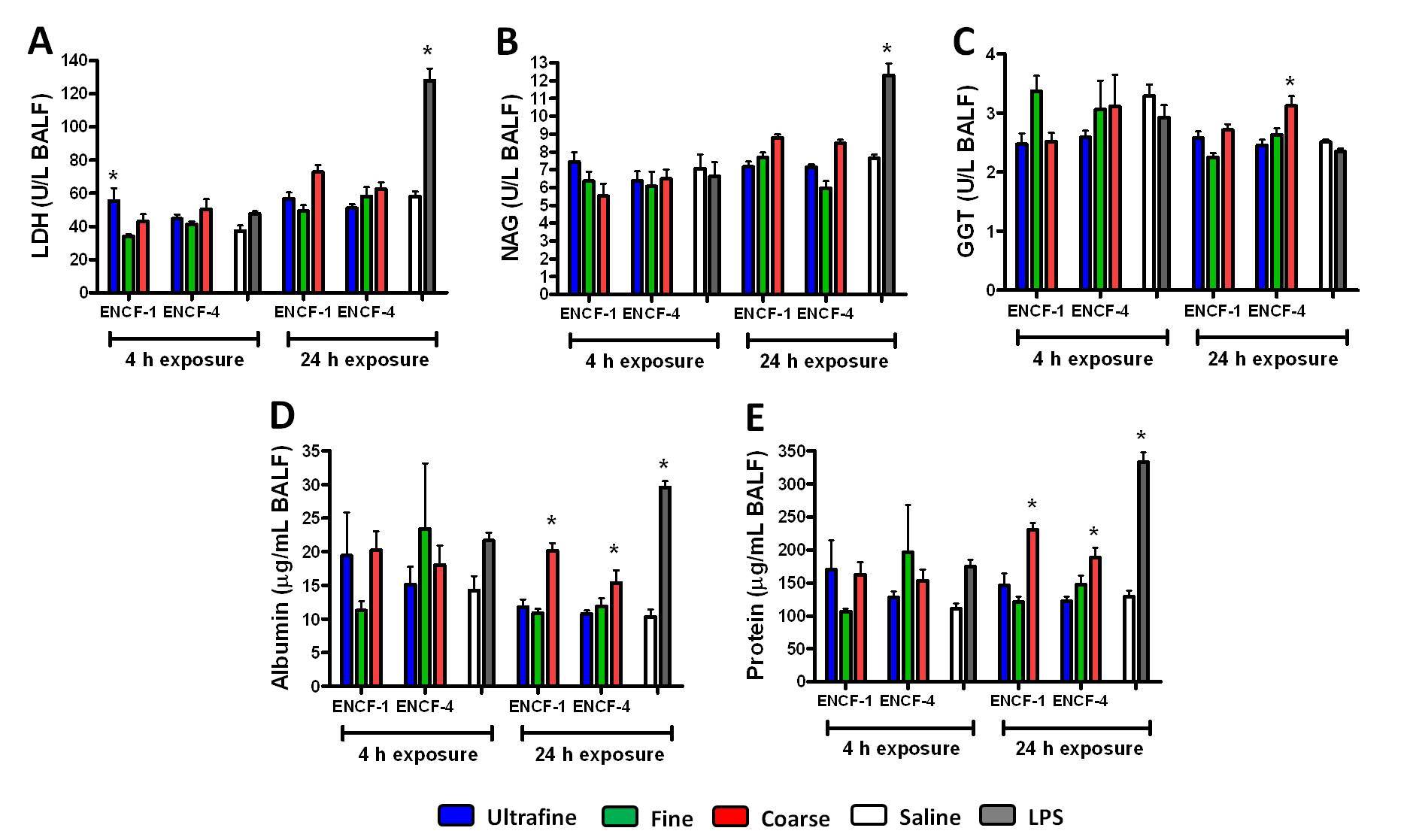

Supplement: Additional file 1: Figure S1 — Biochemical markers for lung injury and edema in BALF of mice at 4 h and 24 h post-exposure to ENCF-1 and ENCF-4 PM (100 μg) by oropharyngeal aspiration. (A) LDH, (B) NAG, (C) GGT, (D) albumin, and (E) total protein concentrations in BALF. Data are means ± SEM (n = 5–6 in each group). *p < 0.05 compared with the saline-exposed negative control group from the same time point. Mice exposed to 2 μg of LPS served as a positive control. [file 1743-8977-11-29-S1.jpeg]

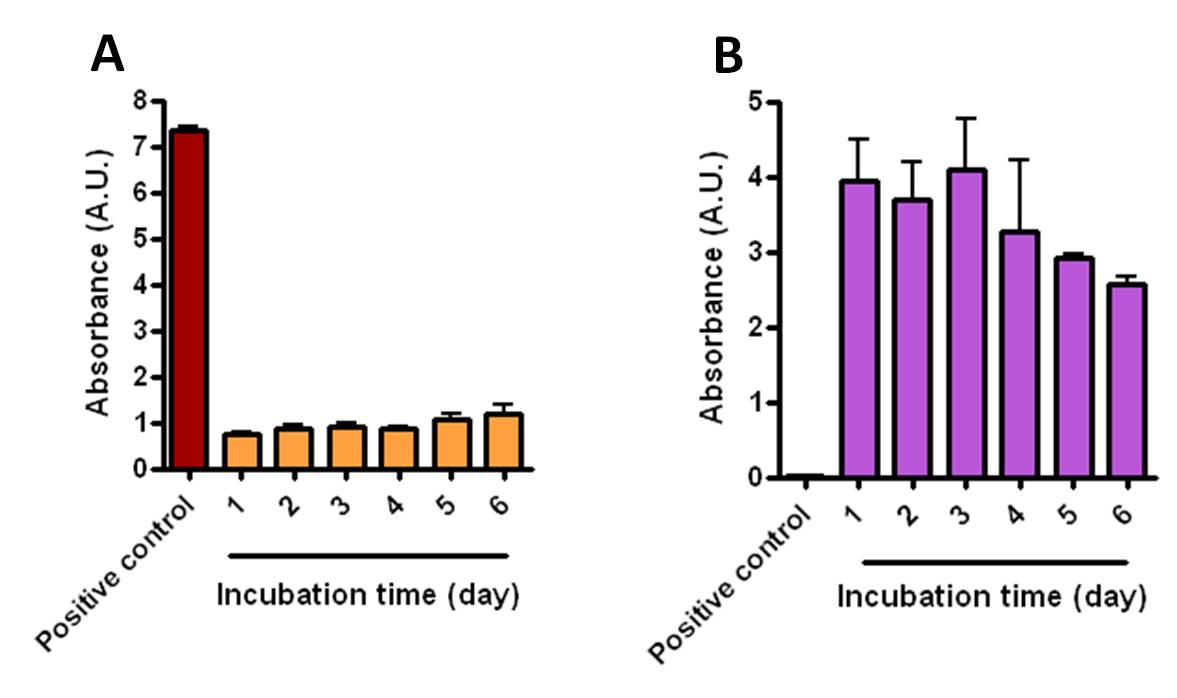

Supplement: Additional file 2: Figure S2 — Viability of lung tissue slices during 6 days of culture. (A) LDH assay and (B) WST-1 assay. Data are means ± SEM (n > 3 in each group). Lung slices exposed to 0.3% Tritron X-100 for 15 min served as a positive control. [file 1743-8977-11-29-S2.jpeg]

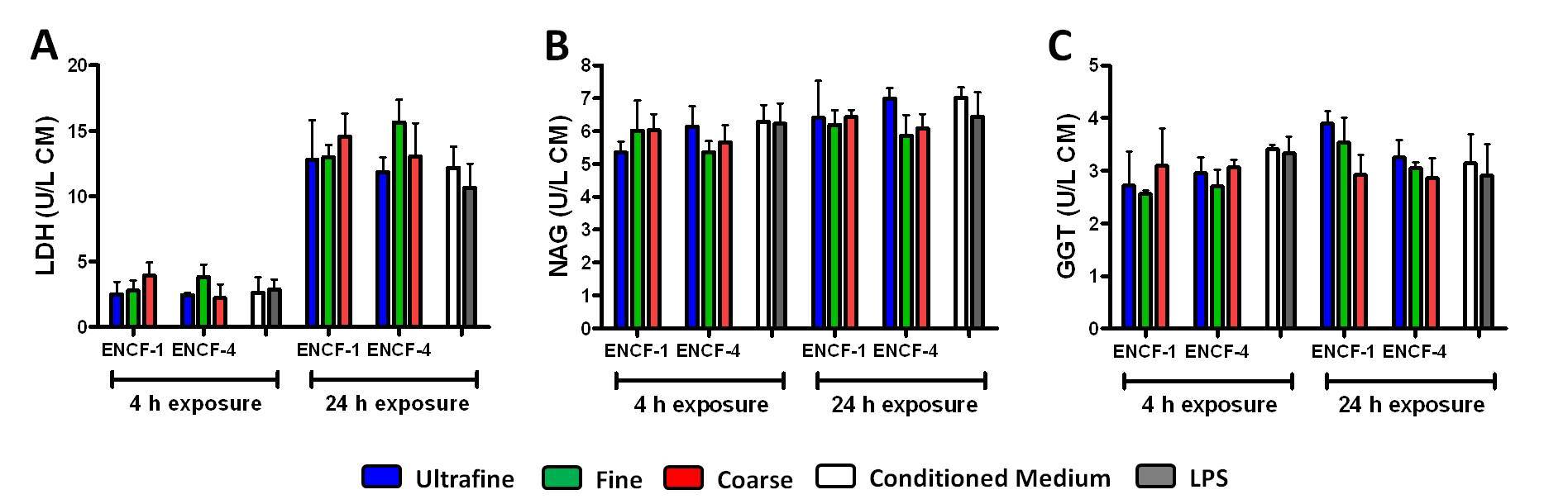

Supplement: Additional file 3: Figure S3 — Biochemical markers for lung injury in lung tissue slices at 4 h and 24 h post-exposure to ENCF-1 and ENCF-4 PM (22 μg/mL). (A) LDH, (B) NAG, and (C) GGT concentrations in the conditioned medium (CM) from the lung tissue slices. Data are means ± SEM (n = 3 in each group). Lung slices exposed to 87 ng/mL of LPS served as a positive control. [file 1743-8977-11-29-S3.jpeg]

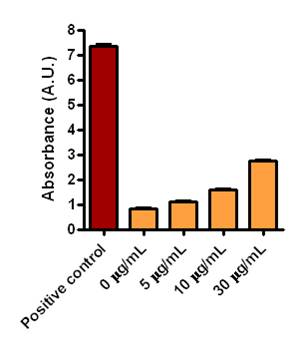

Supplement: Additional file 4: Figure S4 — Dose-dependent LDH release in lung tissue slices exposed to polymyxin B at 24 h. Data are means ± SEM (n > 3 in each group). Lung slices exposed to 0.3% Tritron X-100 for 15 min served as a positive control. [file 1743-8977-11-29-S4.jpeg]

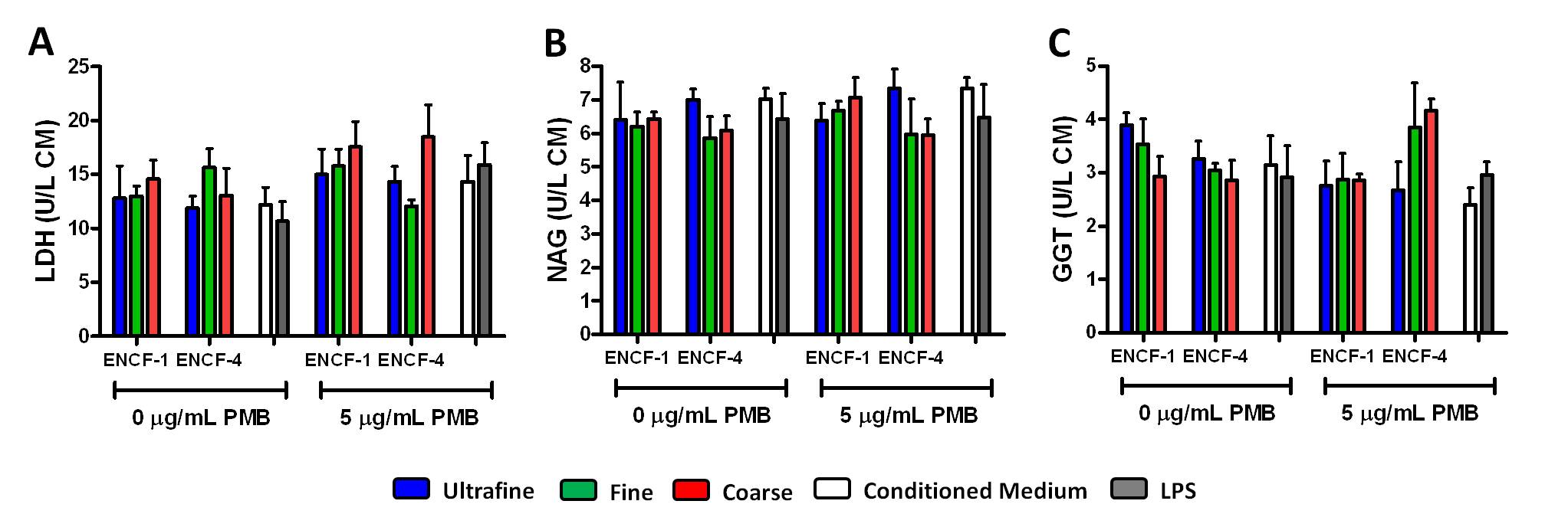

Supplement: Additional file 5: Figure S5 — Biochemical markers for lung injury in lung tissue slices at 24 h post-exposed to ENCF-1 and ENCF-4 PM (22 μg/mL) with and without pre-treatment of polymyxin B (PMB). (A) LDH, (B) NAG, and (C) GGT concentrations in the conditioned medium (CM) from the lung tissue slices. Data are means ± SEM (n = 3 in each group). Lung slices exposed to 87 ng/mL of LPS served as a positive control. [file 1743-8977-11-29-S5.jpeg]
